# Negative Differential Resistance in Conical Nanopore Iontronic Memristors

**DOI:** 10.1021/jacs.4c00922

**Published:** 2024-05-02

**Authors:** Ruoyu Yang, Yusuff Balogun, Sarah Ake, Dipak Baram, Warren Brown, Gangli Wang

**Affiliations:** Department of Chemistry, Georgia State University, Atlanta, Georgia 30302, United States

## Abstract

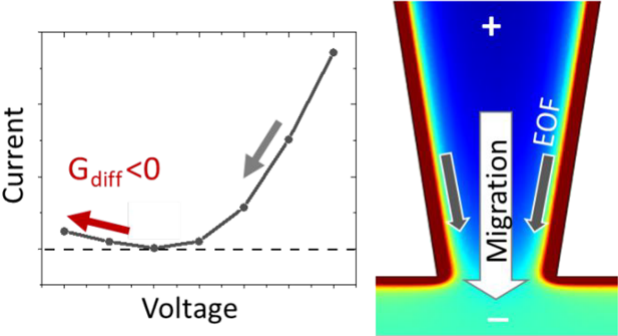

Emerging ion transport dynamics with memory effects at nanoscale solution–substrate interfaces offers a unique opportunity to overcome the bottlenecks in traditional computational architectures, trade-offs in selectivity and throughput in separation, and electrochemical energy conversions. Negative differential resistance (NDR), a decrease in conductance with increasing potential, constitutes a new function from the perspective of time-dependent instead of steady-state nanoscale electrokinetic ion transport but remains unexplored in ionotronics to develop higher-order complexity and advanced capabilities. Herein, NDR is introduced in hysteretic and rectified ion transport through single conical nanopipettes (NPs) as ionic memristors. Deterministic and chaotic behaviors are controlled via an electric field as the sole stimulus. The NDR arises fundamentally from the availability and redistribution of the ionic charges during the hysteretic and rectified transport at asymmetric nanointerfaces. The elucidated mechanism is generalizable, and the drastically simplified operations enable tunable state-switching dynamics with higher-order complexity besides the first-order synaptic functions in multiple excitatory and inhibitory states.

## 1 Introduction

Iontronics and circuit elements with memory effects, e.g., memristors, have attracted significant research interest recently as they could emulate biological neural systems and have the potential to outperform traditional solid-state-element-based systems in applications such as neuromorphic computation.^1−3^ Iontronics refers to the electronics hardware and logic operations based on the emerging properties of solution ion transport confined in nanometer-scale interfaces.^4−9^ Its development is inspired by the information transduction and processing abilities of the central nervous systems, which can express over 20 dynamical behaviors in response to electrochemical stimulation.^10,11^ Compared to the solid-state materials and devices that constitute majority of recent advances in memory devices, iontronics itself and ionic memristors are in infancy and fast growing.^11−14^ Memristors, among other circuit elements with memory effect, are resistors with memory that dynamically adjust their conductivity based on past states under external stimuli.^15−17^ With their unique capability of integrated data storage and information processing, a single memristor can effectively replace multiple transistors on a chip, mitigating the needs of data exchange between logic process and information storage and thus enhancing energy efficiency and computing capacity.^18−20^ Significant challenges remain to be addressed, for example, the first-order complexity problem of the consistent accessibility of multiple states, e.g., the short-term and long-term memory, which in biological systems arise from the adaptation of internal states responding to the environment and history. More advanced functions require controllable dynamics directly coupled with those states, referred to as second- and higher-order complexity problems. New circuit elements and logic functions are prerequisites to solving sophisticated tasks such as ultrafast input integration and pattern classification among other needs to advance iontronics applications and neuromorphic computing beyond von Neumann architecture limitations.^18−20^

Negative differential resistance (NDR) is a rather unique property for electronics in that the conductance decreases with an increase in applied potential. Impressive progresses have been achieved recently in solid-sate systems.^18,19^ To achieve NDR function, complex designs in materials and/or measurement system are introduced in nanopore and nanofluidics studies, including external symmetry-breaking gradients (solvent, concentration, pressure, etc.)^21−25^ and chemical modifications.^26,27^ Our understandings on the hysteresis effects in the rectified charge transport properties in single nanopores lay the foundation to introduce NDR into the iontronics development.^13,28^ Next, we show that the NDR can be conveniently initiated and regulated solely by tuning the local environment in the nanopore region through an applied electrical potential waveform. This is fundamentally significant in that a single stimulus induces multiple variables, i.e., second- or higher-order complexity, closely associated with the phenomenological conductivity features including diode type current–potential (*I–V*) curve and pinched time- and history-dependent *I–V* loops through which we elucidate mechanistic insights for generalization. It is also of technological importance because it eliminates the need for additional variables such as concentration, solvent, and/or pressure gradients,^21−25^ not only allowing for drastically simplified implementation but also enabling sustainable and improved functions. The intricate governing mechanism is elucidated through finite element simulations, which may account for the challenges to discover and control this exciting property in earlier work.

## 2 Results and Discussion

### 2.1 Overview of NDR with Respect to ICR and Memory Effects

**Figure 1:**
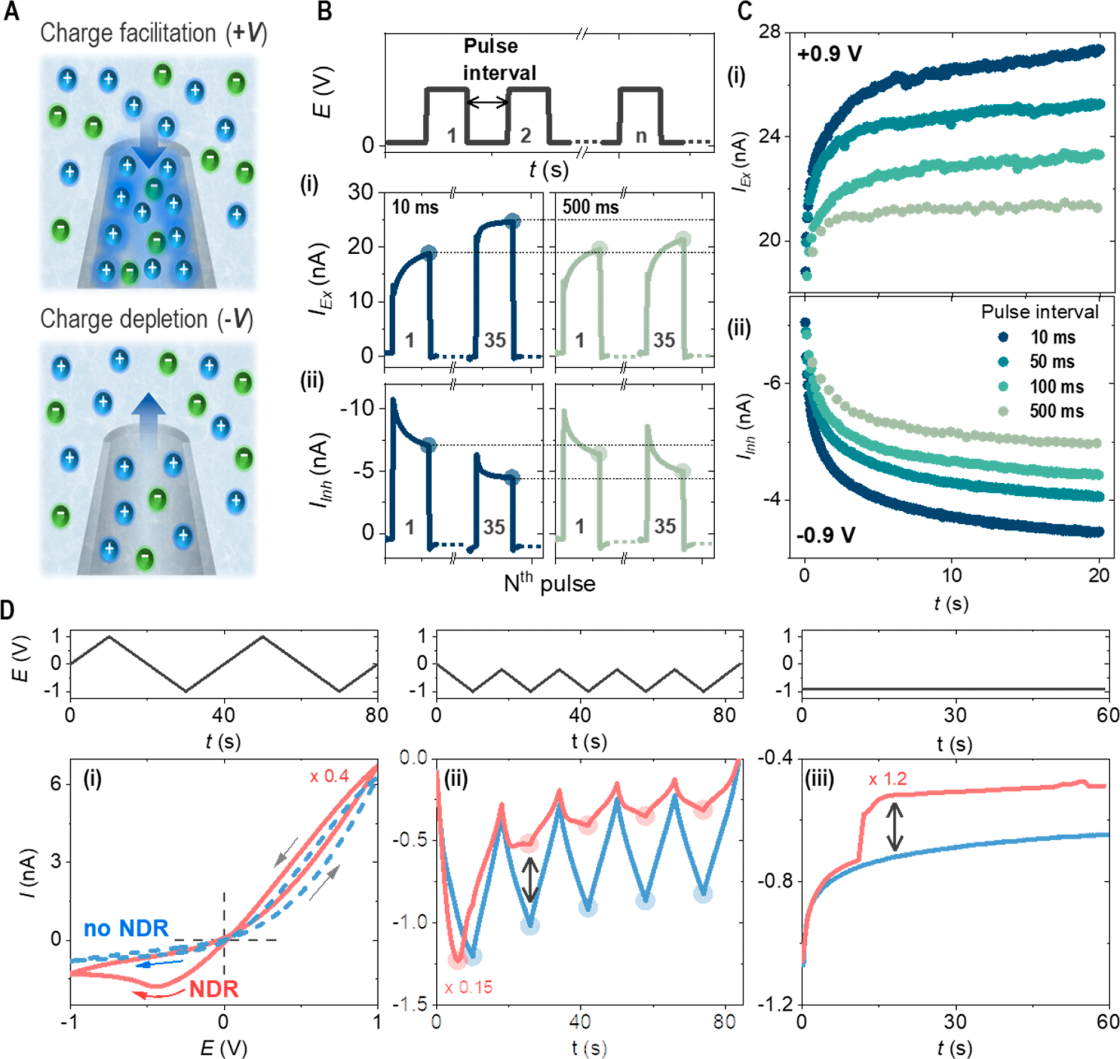
Hysteresis and NDR in rectified ion transport through single nanopipettes. A potential waveform is applied to an Ag/AgCl wire as working electrode in the outside KCl solution against another Ag/AgCl wire as the reference/counter electrode inside the nanopipette containing the same KCl solution. (A) Schematic illustration of the potential bias-regulated facilitation and depletion of charges at a single conical nanotip. (B) Current responses from the 1st and the 35th pulses under voltage pulse train (i: +0.9 V; ii: −0.9 V over duration *t*_p_ = 50 ms) with a pulse interval of 10 and 500 ms through a single 20 nm radius nanopipette in 100 mM KCl solution. (C) Excitatory (i) and Inhibitory (ii) *I–t* profiles under voltage pulse train with different pulse intervals from 10 to 500 ms. The last data point from each pulse response (highlighted by circles in panel B) is plotted in panel C. (D) *I–V* (i) and *I–t* (ii and iii) curves with NDR (red) and without NDR (blue) under triangle waves and constant potentials (−0.9 V) through three ca. 150 nm-radius nanopipettes in 4 mM KCl solution. Current was scaled by the listed factors for an easier visual comparison. Single arrows in panel i indicate potential scan directions. Circles in panels ii and double arrows in panels ii and iii highlight the drastic differences before and after NDR at the same potentials.

Characteristic ionic current features of (1) ion current rectification (ICR), (2) memory effect or hysteresis, and (3) NDR are summarized in Figure 1. The enriched and depleted mobile charges, *Q*_En_ and *Q*_Dep_, in Figure 1A establish high and low conductivity states (HC/LC) of the ICR and hysteresis (Figure 1Di). Those are stimulated by programming the triangular potential waveform across a conical nanopore (tip radius ranging from 20 to 200 nm in suitable ionic strength, discussed later). Memory effects are further illustrated as the excitatory and inhibitory current features in Figure 1B,C (from 0 V to i*:* + 0.9 V to ii*:* −0.9 V). Under potential pulse trains, repeated 50 ms voltage pulse stimulations generate continuous buildup or depletion of mobile charges within each pulse (e.g., #1 or #35) and over repeated pulses (e.g., from #1 to #35), whereas the extent of the changes decreases with the increase in pulse interval from 10 to 500 ms. In other words, the current responses under a shorter pulse interval have a longer effective time for enriching or depleting charges and thus generate a stronger memory effect. Although not the primary focus of this work, these hysteretic ion transport behaviors of a nanopore successfully generate synaptic short-term plasticity, and the insignificant loss over increased pulse interval suggests the feasibility for long-term memory effect.^1,3^ These physical transport processes are intrinsically stable, demonstrating the high accessibility and tunability of multiple conductivity states. Extraordinary reproducibility is anticipated until the enriched or depleted charges alter the bulk concentration (e.g., for a 100 mM 0.1 mL volume, a 10 nA current would require 10^8^ s of continuous stimulation or 10^10^ cycles).

NDR features are highlighted in Figure 1D in amperometry under constant or varied potentials, e.g., triangle potential waveforms in cyclic voltammetry (CV). Compared to rectification and hysteresis, which correspond to diode-type higher/lower current amplitudes and pinched *I–V* loops at opposite potential polarities,^14,29,30^ a system with NDR displays an abrupt or gradual drop in conductance and switches to much lower conductivity states if the applied potential is over a threshold range. Contrary to requiring external additional symmetry-breaking mechanisms,^21−25^ striking NDR features are produced directly using the same 1:1 ionic solution across the nanopipette. This corresponds to a single second- or higher-derivative equation versus two or more first-order derivatives. The switching in conductivity states along with the rectification and hysteresis is significant in that it enables the development of advanced electronic functions at higher-order complexity in iontronics driven by a single stimulating input.

NDR property is built on the rectified ion transport properties and the “memory” of the previous conductance states, both of which are limited by the most restrictive nanotip region. Governed by electrostatic interactions with negative surface charges from the deprotonated silanols on quartz, the mobile charge carriers are mostly cations, which will be the focus of discussion. The negative surface charges induce the electrical double layer (EDL), whose strength is well-known to vary with the ionic strength, surface charge density, and nanopore geometry. Importantly, the direction of the surface electrical field remains constant, normal to the surface, and is separated from the cross-nanopore flux by the half cone angle. The electrostatic forces exerted to the ion transport establish the hysteric effects, characterized by a nonzero cross point potential as explained in our previous reports.^13,30^ Briefly, under a positive applied potential across the nanopore (inside vs outside), cations migrate inward the nanopore also inducing the electroosmotic flow (EOF), a process facilitated by the surface electrical field to enrich charges in the nanopore region.^31,32^ Conversely, applying a negative potential induces an outward cation migration and EOF and depletes the charges. Not just establishing the HC or LC state, the surface field continually enriches ions in HC and causes charge depletion in LC regardless of the increase or decrease of the applied potential magnitudes, inducing the hysteric effects. Thermodynamically, those enriched charges will need to be depleted when the potential bias is stepped or scanned from HC to LC conditions, triggering NDR behavior at a certain potential range, depending on the relative dynamics of the charge redistribution and the stimulus potential.

### 2.2 Kinetics Effects and a Diagnostic Signature of *Q*_LC_/*Q*_HC_

**Figure 2:**
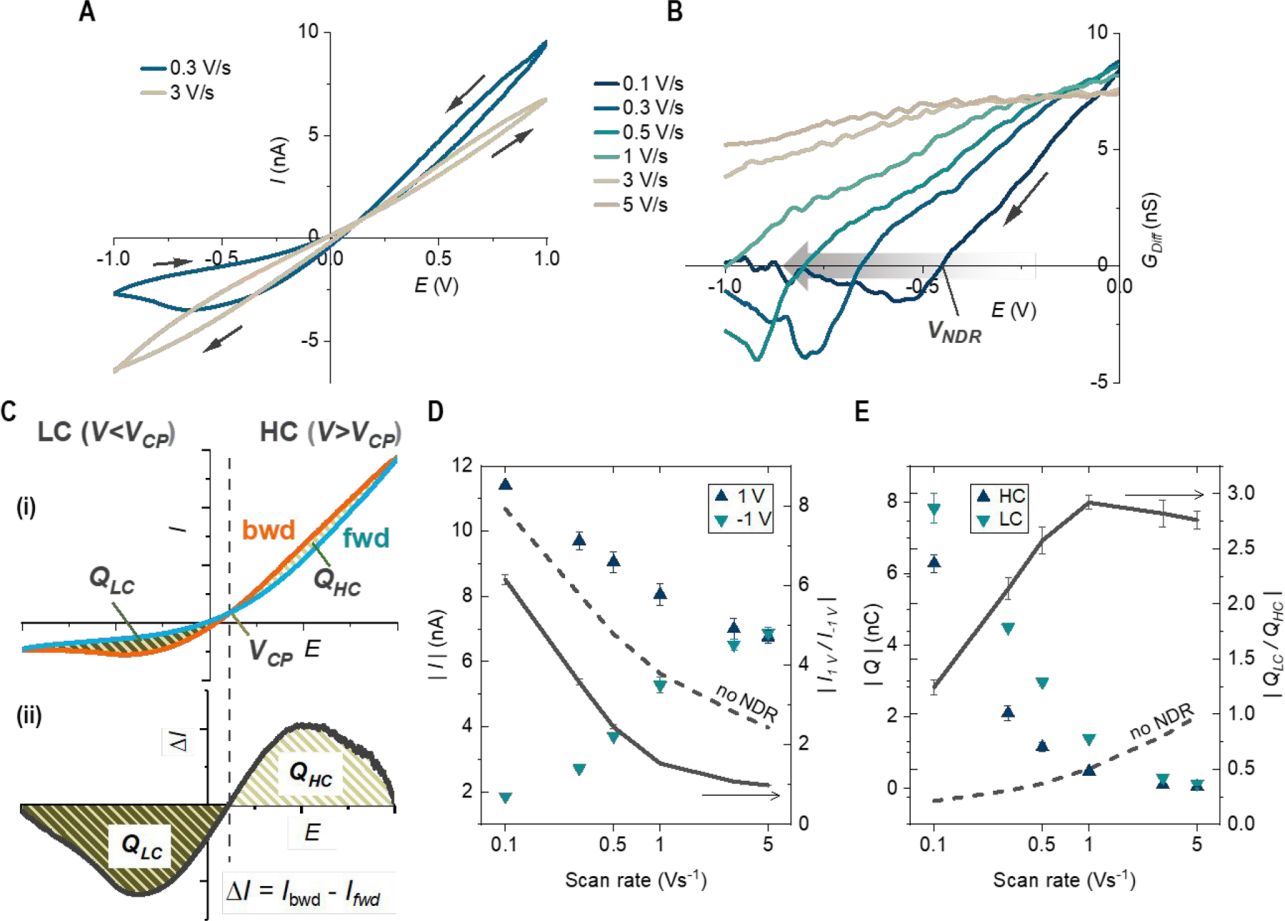
NDR threshold potential and charge analysis. CV data from a 50 nm-radius nanopipette in 4 mM KCl. (A) *I–V* curves with different scan rates (0.3 and 3 V/s shown). Arrows indicate potential scan directions. (B) Differential conductance (*G*_Diff_) of the backward scans (from positive to negative potentials). (C) *Q*_HC_ and *Q*_LC_ within a CV scan. *I*_fwd_ and *I*_bwd_: the current from −1 to 1 V or from 1 to −1 V potential scans; *V*_CP_: cross-point potential; *Q* = *S*_area_/υ, where *S*_area_ is from the current loop area and υ is the scan rate. (D) Absolute current and ICR ratio (*I*_+1 V_*/I*_–1 V_) and (E) absolute hysteresis charges and *Q*_LC_/*Q*_HC_ over different scan rates (in log scale). Symbols and lines for the left and right *y* axis, respectively. Dashed lines represent corresponding ratios in those results without NDR. Full plots, additional scan rates, and *G*_Diff_ of the forward scans are in Figure S1.

NDR dynamics is revealed through analyses of the hysteretic *I–V* curves in Figure 2. At slower scan rates, e.g., 0.1–1 V/s, NDR is consistently observed in the backward potential scan toward LC states after the ion enrichment in the HC states. The NDR peak potential *E*_NDR_, where the differential conductance *G*_Diff_ = 0, increases with scan rate. NDR is no longer observed at about >1 V/s because (1) we limit the LC potential to −1 V to mitigate the possibilities of nanobubble formation from water splitting^33−35^ and (2) the capacitive charging and discharging of the glass membrane substrate superimpose with the measured transport current and shift both the cross potential and the current curvature.^36^ Note that *G*_Diff_ (slope of an *I–V* branch) is more informative over static conductivity because of the memory effects.

NDR corresponds to the transition to the thermodynamically equilibrated steady states through releasing the kinetically enriched charges. The net enriched and depleted charges within a complete HC and LC cycle can be determined from eq 1 as illustrated in Figure 2Cii*.*^28^
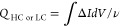
1

These hysteresis charges are the physical origin of the conductivity differences enabling the NDR. The analyses in Figure 2D,E shed light on the relationship of NDR with ICR and hysteretic charges. Increasing the scan rate reduces the time for charge polarization. Consequently, both HC and LC hysteresis charges decrease. Less charge polarization also leads to less rectification; i.e., the ICR ratio approaches 1 when HC current decreases and LC current increases with scan rate.^37,38^ NDR diminishes accordingly. The trend of ICR features is qualitatively similar to with or without NDR (solid vs dashed). However, NDR is more sensitive to the hysteric charges and generally associated with larger *Q*_LC_ or *Q*_LC_ > *Q*_HC_. A clear distinction is the ratio of hysteretic charges, *Q*_LC_/*Q*_HC_, which reaches a maximum at the intermediate scan rates. In comparison, those without NDR tend to have much lower *Q*_LC_, with the *Q*_LC_/*Q*_HC_ (<1) increasing monotonically with scan rate to approach unity. These trends are further shown in Figures S2 and S3.

To corroborate with the complexity described by differential equations,^18^ the through-nanopore ion flux and the hysteresis charges are two variables both affected by the applied and surface electrical fields. Further complicating the analysis, the surface field can vary under experimental conditions depending on the deprotonation reactions,^39,40^ and anion transport may not always be negligible under all conditions. Therefore, NDR corresponds to at least second-order and likely higher-complexity properties.

### 2.3 Active Controls on NDR Switchings: Gradual and Abrupt

**Figure 3:**
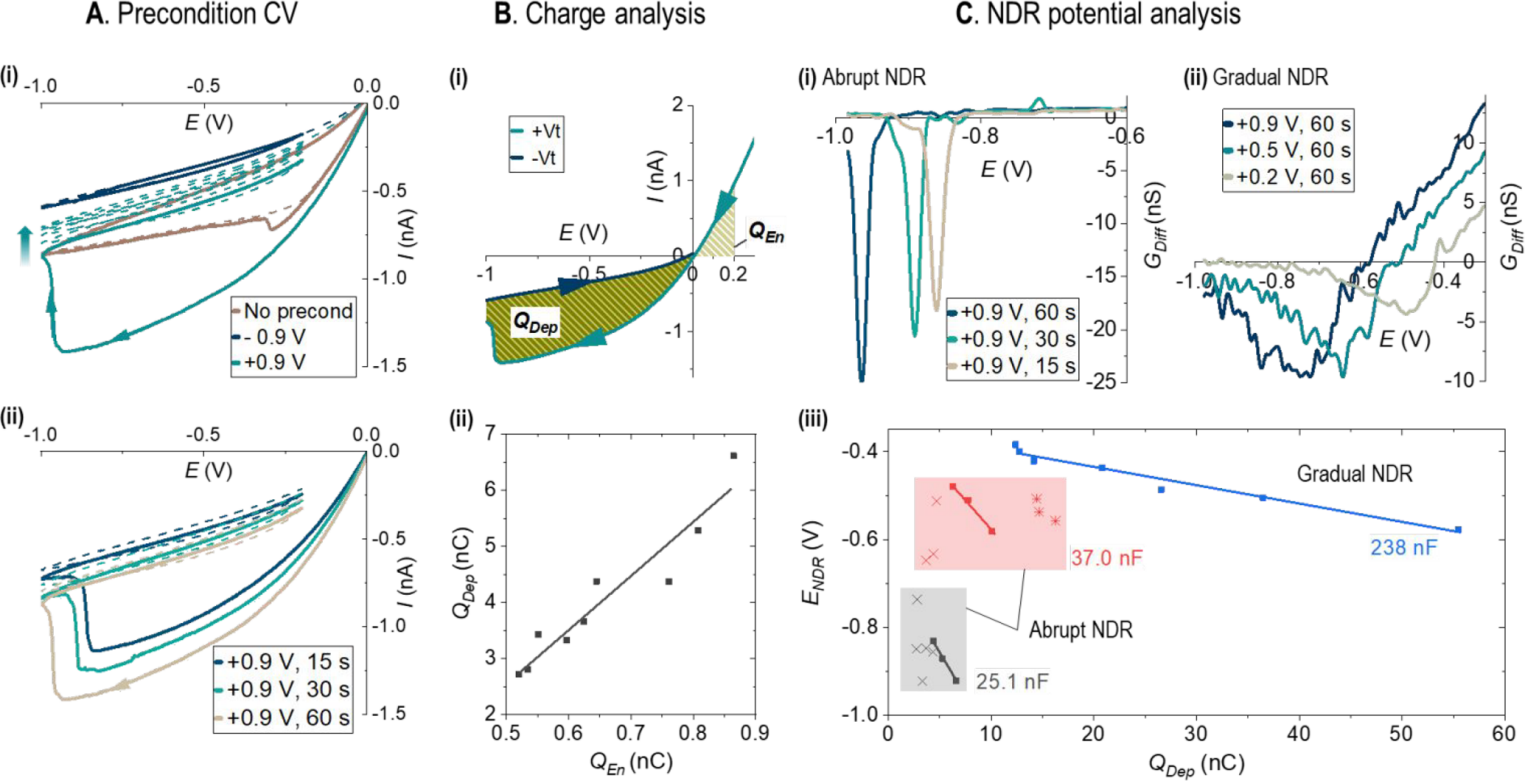
NDR tunability by controlling the precondition potential and time. Data from a 100 nm-radius nanopipette in 4 mM KCl solution at 0.1 V/s. (A) Impacts of precondition potential and duration on NDR. (i) LC current without and with constant precondition potentials at +0.9 and −0.9 V over 60 s. Arrow indicates the current decrease of the +0.9 V data over time/scans. (ii) Varied precondition durations under constant enrichment potential at +0.9 V. The potential was scanned negatively and limited within [−0.2, −1.0 V] after preconditioning. The first scan is plotted as a solid line with the following scans as a dashed line (two cycles shown). (B) *Q*_En_ and *Q*_Dep_ in the presence of the NDR effect. (i) Calculations of charges. *Q*_Dep_ = *S*_Dep_/υ; *Q*_En_ uses the areas within +0.2 to 0 V for simplicity as explained in the SI. (ii) *Q*_Dep_ after nine different enrichments under +0.2/0.5/0.9 V over 15/30/60 s, respectively. (C) Gradual and abrupt NDR switching characterized by *E*_NDR_–*Q*_Dep_ relationship. (i) Abrupt and (ii) gradual *G*_diff_ curves after ion enrichments. (iii) Zone diagram. Intrinsic and preconditioning CVs are in Figures S4–S7.

Convenient controls of the NDR properties are demonstrated in Figure 3 by tuning ion enrichment. The results directly illustrate the adaptive behaviors that control state-switching to ultimately enable temporal dynamics for higher-order complexity functions. Compared to the intrinsic NDR in Figure 3A, enrichment preconditions enhance NDR, e.g., a constant bias at +0.9 V over 60 s (i) and varied durations (ii), or a depletion precondition suppresses it entirely. Quantitated in Figure 3B, a linear relationship can be seen between the NDR-released charges and the enrichment that is described by the *Q*_En_ charges under the highlighted area (validation in Figures S8 and S9). Mechanistically, a higher potential or a longer time increases the level of enrichment prior to NDR switching, i.e., the initial NDR states, thereby strengthening the NDR. To avoid reentering a charge facilitation state that would complicate the analysis, subsequent potential scans after preconditioning are restricted within the range of −0.2 to −1.0 V. NDR is thus present primarily in the first scan cycle. Accordingly, *Q*_En_ is used to characterize the enrichment preconditioning, as no complete HC *I–V* loop is available to calculate *Q*_HC_. The ratio of *Q*_LC_/*Q*_HC_ or *Q*_Dep_/*Q*_En_, linearly correlated in Figure S9, is believed to indicate the intrinsic capability of individual NPs that require systematic studies to correlate with the intricate nanogeometry and surface charge parameters.^41−43^

Two types of NDR switching are observed in different NPs or after varied enrichments: abrupt and gradual. As illustrated in Figure 3Ci,ii, the *G*_Diff_ reaches a maximum at about 20 nS and stays negative over about 0.5 s in an abrupt NDR versus about 5 nS over 5 s in a gradual transition. The distinction is more eminent at second-order derivatives, i.e., the rate of d*G*_diff_/d*t*, over the stimulation amplitude or duration individually. Generally, the gradual NDR features are observed mostly from NPs with higher current amplitude, larger hysteretic charges, and intrinsic NDR. We then define the specific capacitance of NDR based on the good linear *E*_NDR_–*Q*_Dep_ relationship in eq 2:
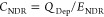
2

In gradual NDR, a single continuous event releases most hysteric charges to approach the thermodynamically stable conductance. This final NDR state is characterized by the threshold of the most depleted LC state after −0.9 V of preconditioning. The *C*_NDR_ is about 10-folds larger than those with abrupt multistep NDR transitions. In abrupt NDR, multiple minor switching events occur over multiple scans, generally from those with lower current amplitude and/or hysteretic charges (Figure S5 and S6). Regardless of the initial states after enrichment or the intermediate steps, the ion current ultimately approaches the same threshold LC state (i.e., after the −0.9 V preconditioning) for a given nanodevice-solution system. The minor and incomplete switching events reveal intermediate metastable states. With less enrichment or lower *C*_NDR_, we speculate that a smaller energy difference is less effective in driving a single or monotonous transition. Instead, multiple switching events appear in a range of potentials and times that result in the stochastic distributions of the *E*_NDR_. This chaotic behavior represents high-order complexity that is potentially useful for applications such as random number generation and data encryption in addition to the construction of logic functions in iontronics.

Within the abrupt NDR zones, however, a single major NDR transition can still be stimulated with an appropriate enrichment. In one example where the NP does not have intrinsic NDR (gray zone), the data from the three major NDR ones in Figure 3Aii are bolded from which the *C*_NDR_ is determined to be 25 nF. Other conditions with less enrichment cause multistep intermediate transitions, some occurring in later *I–V* cycles with weak current decreases at random potentials/times (Figures S5 and S6). In the next example where the NP has intrinsic NDR (red zone), abrupt instead of gradual transitions are observed. The *C*_NDR_ is found to be 37 nF from those with a single major NDR transition. This data set represents the transition between the intrinsic gradual and the induced abrupt NDR zones. Whereas those lower *Q*_Dep_ trails (denoted “X”) trigger stochastic switching, we believe that the variations among the three higher *Q*_Dep_ (“*”) are within experimental error, likely reaching threshold enrichment limited by the effective volume at the nanotip.

### 2.4 Transport Pathways Revealed by Finite Element Simulation

**Figure 4:**
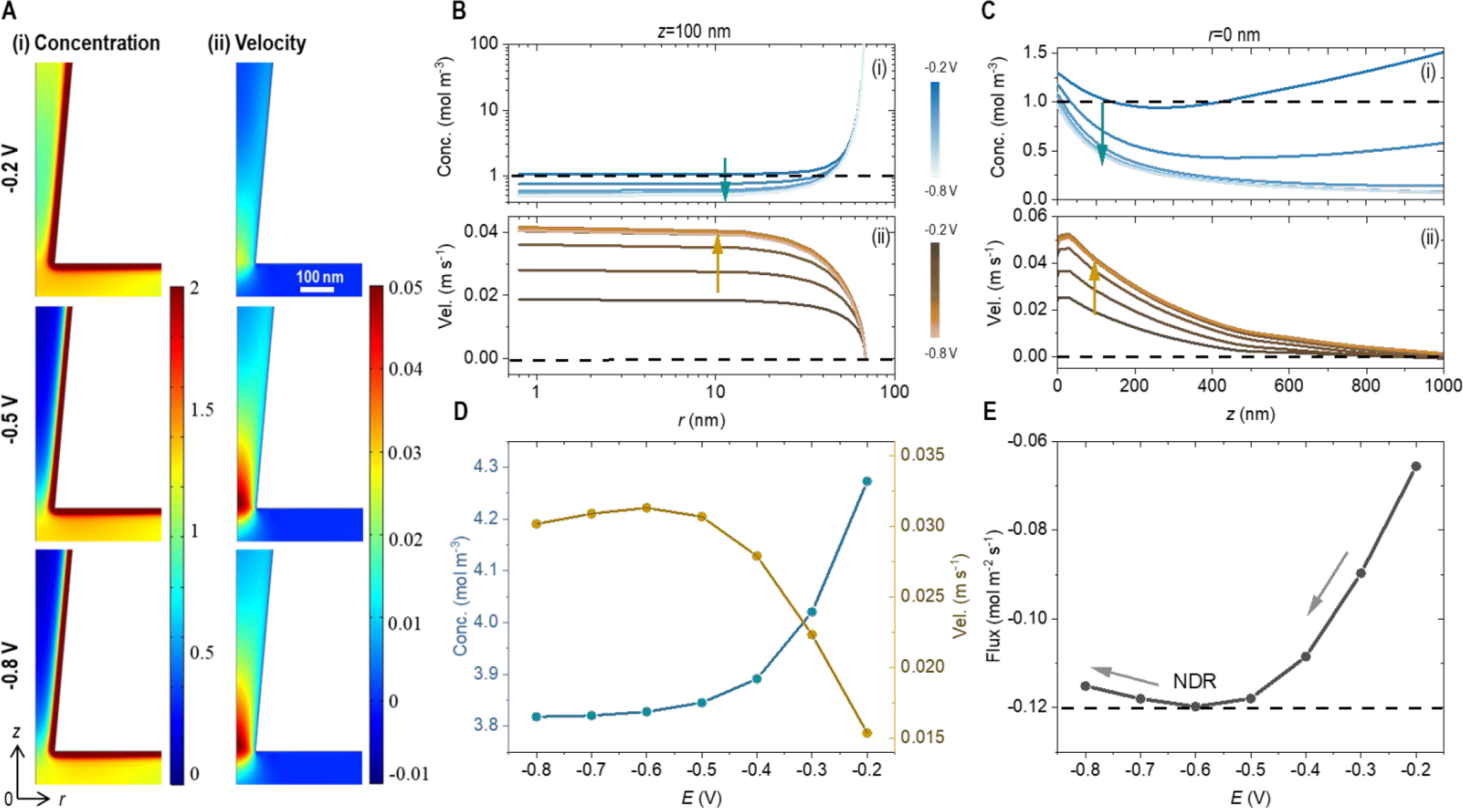
Origin of NDR is illustrated by the simulated K^+^ concentration and velocity profiles. The data are from the backward current displaying NDR features using a 60 nm-radius conical pore and a half cone angle of 5° in 1 mM KCl solution by solving Poisson and Nernst–Planck coupled with Navier–Stokes equations. (A) Two-dimensional [*r*, *z*] profiles of (i) K^+^ concentration and (ii) EOF velocity at representative potentials before, at the peak, and after NDR transition. Half of the nanopore cross section is shown. Radial length *r* = 0 corresponds to the nanopore centerline in the *z* direction, whereas *z* = 0 is the nanopore orifice. Details of the simulation are in the SI. (B) Radial *r*- and (C) centerline *z*-directional distributions of concentration (i) and velocity (ii). Arrows indicate the potential increase from −0.2 to −0.8 V. Dashed lines indicate zero bulk concentration and velocity. Note that cations are significantly enriched toward the negatively charged side wall and plotted in log scale in panel Bi. (D) K^+^ concentration and EOF velocity at the cross-section *z* = 100 nm (averaged along *r*) at different potentials and (E) the corresponding flux profile that successfully reproduces the experimental NDR feature. Corresponding Cl^–^ data are much smaller/insignificant and plotted in Figure S10.

Migration and EOF collectively govern the NDR behaviors, as revealed in Figure 4. The K^+^ concentration and EOF profiles at representative [*r*, *z*] locations highlight the intrinsic inhomogeneous distributions at the transport-limiting conical nanopore region under bias potentials around the NDR transition. Without external concentration gradient(s) or pressure differential, cation migration and associated EOF are the main contributing ion transport mechanisms, which are further confirmed in Figure S10.^24,44,45^ Near the negatively charged surface, cation concentration is very high, whereas the EOF velocity is zero at this nonslip boundary. Along the centerline, the cation concentration is lower than the bulk concentration in most nanopore regions, from the orifice up to several microns inside and then back to the bulk concentration toward the nanopore base (not shown), where surface effects become insignificant. EOF is mostly within the surface EDL, so its velocity at the centerline decreases to negligible within about 1 μm inside the conical nanopore. Higher potentials further deplete the cations, the main charge carrier, and extend the depletion volume. Meanwhile, a stronger applied potential tends to drive higher EOF velocity, which is carried by cations as charge carriers, i.e., dependent on cation availability. The intricate interplay between migration and EOF under different potentials results in the crossover in Figure 4D. Those averaged profiles along the cutline correspond to the integrated flux at the cross section in Figure 4E that reproduces the NDR features successfully. Therefore, the NDR results from the opposite dependence of ion concentration and EOF velocity on the polarization potential, and the intrinsic gradient distributions of ion concentration and EOF are adequate to regulate NDR, eliminating the requirements of additional gradient(s) or stimulating mechanisms.

Simulation mitigates experimental uncertainties such as the heterogeneity of individual nanodevices without concerns on device fractures or surface charge variations for systematic studies. The convoluted impacts by nanogeometry such as radius and cone angle on ion transport current at different ionic strengths are revealed in Figure S11. Because symmetric nanofluidic devices will not have rectified or selective transport properties without symmetry-breaking factors, neither memristor hysteresis nor NDR is expected in cylindrical nanochannels, i.e., zero cone angle. A smaller half cone angle of 5°, however, is found to induce NDR, but not a 15° angle under otherwise identical conditions. Also, intermediate sizes, about tens of nanometers, generate stronger NDR over larger or smaller counterparts. The trend is qualitatively consistent with the competition mechanism between concentration polarization and EOF; the combined effect strengthens at intermediate sizes and ionic strength as explained in our earlier reports.^31,45^ Although we believe that the mechanism and the trend are applicable to other nanofluidic devices, the continuum theory-based simulation results should only be taken as a general guideline and not quantitatively for fitting. For example, it is not uncommon in experiments to observe strong NDR in tens of millimolar ionic strength or higher from NPs with a radius more than 100 nm, which is highly favorable for applications (Figures S12 and S13). However, in simulation, ICR, hysteresis, and NDR can only be observed at lower ionic strength of about 0.1–10 mM.

It is worth emphasizing that the elucidated ion transport dynamics responsible for the NDR is often a pivotal physicochemical process in membrane- or porous-material-based ion separation and various electrochemical systems. Therefore, the fundamental understanding and the NDR properties provide new solutions and pathways to many challenges, for example, the precondition ion enrichment and the selective ion transport to mitigate the trade-off in separation selectivity and throughput, to improve the efficiency in energy harvesting from salinity gradients, and to deconvolute the coupled ion transport and electron transfer in many electrochemical reactions.

## 3 Conclusions

NDR properties are successfully generated by exploiting intrinsic concentration and flux gradient(s) localized in single conical nanopores, driven, and tailored simply via an external potential waveform. Two characteristic parameters for NDR capacity are established: the ratio of depleted over enriched hysteric charges and the specific capacitance. Through combined experimental and simulation approaches, the NDR mechanism is determined to arise from the competition between EOF and concentration polarization during nanoscale electrokinetic ion transport. The elucidated mechanism and the drastically simplified operations replicate biological neural functions in accessing multiple excitatory and inhibitory states with extraordinary consistency; generate deterministic and chaotic state-switching NDR properties; provide new design principles for advanced logic operations in iontronics; offer new pathways to mitigate the bottleneck trade-offs of selectivity-throughput in electrokinetic ion separation and energy conversion processes; and are generalizable to other materials and device platforms for iontronics, neuromorphic computing, ion enrichment and separation, and other energy applications.

## Supporting Information Available

Materials and methods; Comsol simulation model details; additional experimental results (Figures S1–S11) (PDF)

## ABBREVIATIONS

NDR: negative differential resistance
NP: nanopipette
ICR: ion current rectification
HC: high conductivity
LC: low conductivity
EOF: electroosmotic flow
