# Supplementary material for: Negative Differential Resistance in Conical Nanopore Iontronic Memristors
Source: J Am Chem Soc. 2024 May 2;146(19):13183–90. doi: 10.1021/jacs.4c00922 (PMC11099999; doi:10.1021/jacs.4c00922)
Supplement: Supplementary file 1 — ja4c00922_si_001.pdf [file ja4c00922_si_001.pdf]

Negative Differential Resistance in Conical Nanopore Iontronic Memristors

Ruoyu Yang, Yusuff Balogun, Sarah Ake, Dipak Baram, Warren Brown[#], Gangli Wang*

Department of Chemistry, Georgia State University, Atlanta, GA, 30302, United States

#: current address: Department of Chemistry, Monmouth University, NJ, 07764, United States

Table of Content

Materials and Methods.

Simulation Details.

Table of Figures:

Figure S1. CV data from a 50-nm nanopipette in 4 mM KCl (original data of Figure 2). (A) I-V curves at different scan rates. The potential scan directions are indicated by the arrows. (B) Differential conductance (G_{Diff}) of the forward scans (from negative to positive potentials). $G_{Diff} > 0$ throughout the potential window means no NDR is observed.

Figure S2. Representative CV, differential conductance, and charge analysis of NPs without NDR. Data from a 200-nm nanopipette in 4 mM KCl. (A) I-V curves with scan rates of 0.3 and 3 V/s. (B) Differential conductance (G_{Diff}) of the backward scans (from positive to negative potentials. (C) ICR and (D) hysteretic charges (<1) over scan rate. Dashed lines are the ratios of ICR and hysteretic charges from the NP with NDR in Figure 2 for comparison, i.e., lower ICR but higher charge ratio (>1).

Figure S3. Consistency in the ratios of (A) ICR and (B) hysteretic charges over scan rate from multiple NPs. Solid lines are ICR and charge ratio from three nanopipettes showing NDR, i.e., lower ICR but higher charge ratio, versus dashed lines from three nanopipettes without NDR. The solid indigo lines and dashed beige lines are used in Figures 2 & S2.

Figure S4. CV data from a 100-nm nanopipette (used in Figures 3A, 3B, 3Ci, and induced abrupt zone) in 4 mM KCl. (A) I-V curves with different scan rates. The potential scan directions are indicated by the arrows. (B) Differential conductance (G_{Diff}) of the backward scans (from positive to negative potentials). No intrinsic NDR was observed without precondition enrichment.

Figure S5. Induced NDR with abrupt transitions. Data from a 100-nm nanopipette (original CV in Fig. S4) in 4 mM KCl solution at 0.1 V/s after the denoted precondition duration and enrichment potential. The sweeping potential was limited with [-0.2 V, -1.0 V] after preconditioning. Arrow indicates the gradual decrease of the +0.9 V, 60 s curve over five scans. Original CV is in Figure S4; Charge and differential conductance analysis are in Figures 3Bii and 3Ci, respectively.

Figure S6. Intrinsic NDR with abrupt transitions. Data from a 50-nm nanopipette in 4 mM KCl solution at 0.1 V/s after the denoted precondition duration and enrichment potential. The sweeping potential was limited with [-0.2 V, -1.0 V] after preconditioning. Original CV and differential conductance analysis are in Figure 2.

Figure S7. Intrinsic NDR with graduate transitions. Data from a 450-nm nanopipette in 4 mM KCl solution at 0.1 V/s after the denoted precondition duration and enrichment potential. The sweeping potential was limited with [-0.2 V, -1.0 V] after preconditioning. Original CV and differential conductance analysis are in Figure S13.

Figure S8. Characterization of the precondition. (A) CV data after the denoted precondition enrichment duration and potential. Data from a 100-nm nanopipette in 4 mM KCl solution at 0.1 V/s (used in Figures 3A, 3B, 3Ci, and induced abrupt zone). (B) Correlation of the conductance at +0.2 V and the enriched hysteretic charges (area under the current curve over scan rate, see Figure 3 for definition). Blue line is the linear regression fitting with R value=0.99. Charge and differential conductance analysis are in Figure 3Bii and 3Ci, respectively.

Figure S9. Correlation of the depleted/enriched hysteresis charge Q_{Dep}/Q_{En} and the LC/HC hysteresis charge loop Q_{LC}/Q_{HC}. Q_{LC} and Q_{HC} are varied by scan rates in the range of 0.1 to 1 V/s (see Figure 2 for definition); Q_{Dep} and Q_{En} is varied by precondition enrichment (see Figure 3 for definition).

Figure S10. Simulated I-V curves through a 60-nm pore and a half cone angle of 5° in 1 mM KCl solution. (A) I-V curve. (B) The contribution of cation and anion to the total flux. (C) The contribution of EOF, migration and diffusion to the total cation flux.

Figure S11. Simulated I-V curves with nanogeometry and measurement parameter variations. The top panels are the simulated I-V curves and the corresponding bottom ones are differential conductance (GDiff) of the backward scans after enrichment. (A) A 60-nm nanopore with different half cone angles in 1 mM KCl; (B) different tip radius with a half cone angle of 5° in 1 mM KCl; (C) a 60-nm-radius nanopore with a half cone angle of 5° in different KCl concentrations.

Figure S12. CV data from a 100-nm nanopipette showing NDR in 50 mM KCl. (A) I-V curves with different KCl concentrations at 1 V/s. (B) I-V curves with different scan rates in 50 mM KCl. Panels (ii) present a magnified view of the box area highlighted in panels (i).

Figure S13. CV data from a 450-nm nanopipette (used in Figure 3Cii and intrinsic gradient zone) in 4 mM KCl. (A) I-V curves with different scan rates. The potential scan directions are indicated by the arrows. (B) Differential conductance (G_{Diff}) of the backward scans (from positive to negative potentials). The potential at G_{Diff} =0 corresponds to NDR peak potential (V_{NDR}).

Experimental:

Materials and methods

Nanopipettes were fabricated from quartz capillaries (O.D.: 1.0 mm, I.D.: 0.70 mm, 7.5 cm length, no filament, Q100-70-7.5, Sutter Instrument Co.) using a P-2000 laser puller (Sutter Instrument Co.). The pulling parameters for ca. **50-nm-radius** nanopipettes are: Heat: 700, Filament: 4, Vellocity:60, Delay:150, Pull: 120 or 155; for ca. **150-nm-radius** nanopipettes: line 1: Heat: 700, Filament: 4, Vellocity:55, Delay:180, Pull: 80, line 2: Heat: 700, Filament: 4, Vellocity:60, Delay:150, Pull: 120 or 150.

The nanopipettes were backloaded using an in-house-made micro-injector constructed with a syringe and PTFE tubing in the sequence of acetonitrile, water and KCl electrolyte. Centrifugation at 5000 rpm for 15 min was performed after each loading. Due to the heterogeneity nature in geometry and surface chemistry in nanopipettes, conductivity characterization with 1 M KCl solution is adopted for size calculation following standard procedures in literature using the equation[1, 2]:

$$R_{cone} = \frac{1}{\pi \Lambda r tan\theta} + \frac{1}{4\Lambda R}$$

In this equation, R_{cone} is the Ohmic resistance of the nanopore; Λ is electrical conductivity of the electrolyte; r is the radius of a nanopipette; θ is the half cone angle, determined to be 3.5° by SEM.[2] In 1 M KCl solution (Λ =10.9 S/m), a linear ohmic *I-V* curve is obtained indicating negligible surface effects and minimal access resistance $\frac{1}{4\Lambda R}$, and thus allows volumetric resistance calculation.

The conductivity data were sampled at 1 mV/step using Gamry Reference 600 (Gamry Co.). The electrical potential was applied through two silver/silver chloride wires, ONE inside (RE/CE) and another outside (WE) the nanopipette containing the same electrolyte solution.

Experimental data treatment

Ion current rectification, hysteresis charge, and enriched/depleted charge analysis used original conductivity data directly without further treatment. For differentiation analysis of G_{Diff}, the current was firstly smoothed by a low-pass FFT filter with points of window=5 and cutoff frequency=100, and further smoothed by Savitzky-Golay method with points of window=20 and polynomial order=2.

Finite element simulations

Finite element modelling is performed with COMSOL Multiphysics v. 5.5 with electrochemistry module by solving the Poisson (equation 1), Nernst-Planck (equation 2), and Navier-Stokes (equation 3) equations. Each equation was selected from the modules in COMSOL for Transport of Diluted Species (Nernst-Planck), Electrostatics (Poisson) and Laminar Flow (Navier-Stokes).[2, 3]

$$\nabla^2 (\varepsilon_0 \varepsilon_r \phi) = -F \sum z_i c_i \qquad (1)$$

ε_0 is the permittivity of free space, ε_r relativative permittivity of the solvent, F is Faraday's constant, z_i is the charge of the ion, c_i is the concentration of the ion, and ϕ is the electric potential. The Poisson equation correlates the electric potential and surface charge density and distribution of the nanopore.

$$J_i = -D_i \nabla c_i - \frac{z_i F}{RT} D_i c_i \nabla \phi + c_i \mathrm{u} \qquad (2)$$

J_i is the flux of an individual ion; D_i is the diffusivity of the ion; R is the gas constant; T is the temperature; and u is the fluid velocity. The Nernst-Planck equation governs ion fluxes and currents by diffusion (first term), electric field (second term) and convection (third term).

$$\mathrm{u}\nabla\mathrm{u} = \frac{1}{\rho}\left(-\nabla p + \eta\nabla^2\mathrm{u} - \left(F\sum z_i c_i\right)\nabla\Phi\right. \quad (3)$$

ρ is the density of the solvent, p is the pressure and η is the viscosity. The third term in the Navier-Stokes equation represents EOF. Pressure differential is zero in this study.

The nanopore structures and boundary conditions are based on experimental results and validated in previous reports.[4-6] The geometry of the simulation structure is shown in Scheme S1. The nanopipette radius (r) and half cone angle (θ) are defined based on experimental results. Length of the structureAB = IJ = 5 µm; BC = IH = 2 µm; Lp = 10 µm (AJ-BC-HI); and DE = FG = 1 µm. A gradient surface charge density is applied with a maximum of 80 mC/m^2 that decays exponentially to a bulk value of 1mC/m^2 justified in previous reports.[4-6] Note a constant SCD or with linear gradient would display the same qualitative trends. The gradient is introduced to accommodate with the impacts on the deprotonation equilibrium of surface silanol groups by the applied electric field.

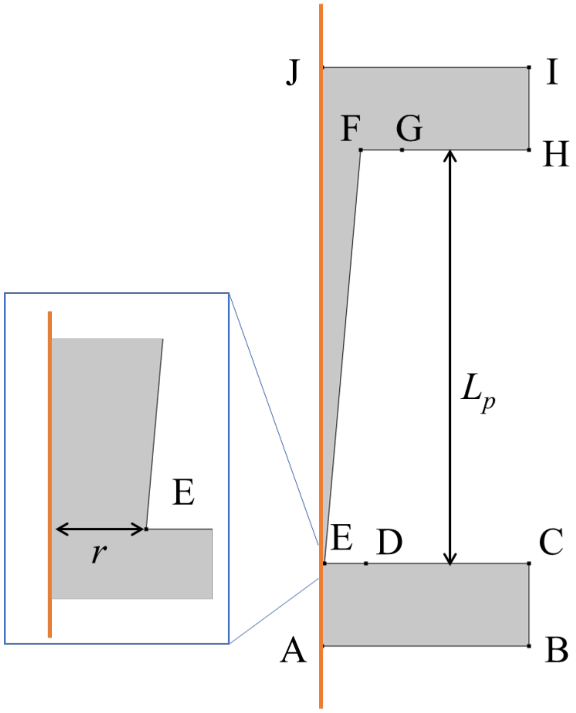

Scheme 1. Simulation Structure. AJ represents the centerline of the nanopore (orange), and the symmetric plane for the axial-symmetric boundary condition.

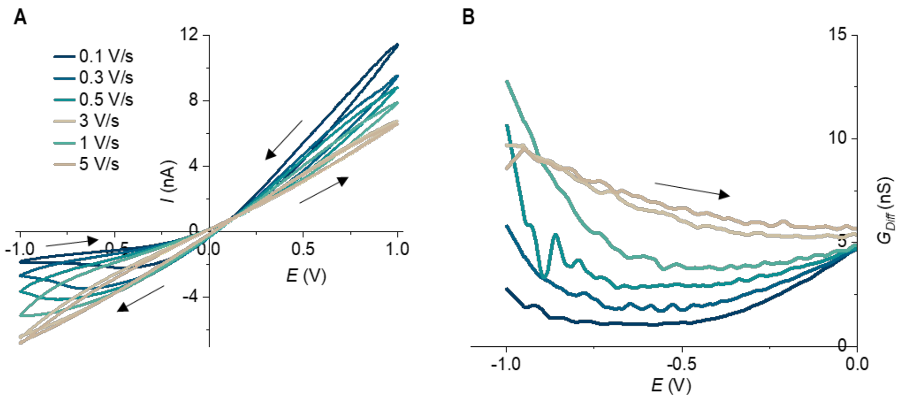

Figure S1. CV data from a 50-nm nanopipette in 4 mM KCl (original data of Figure 2). (A) I-V curves at different scan rates. The potential scan directions are indicated by the arrows. (B) Differential conductance (G_{Diff}) of the forward scans (from negative to positive potentials). G_{Diff} >0 throughout the potential window means no NDR is observed.

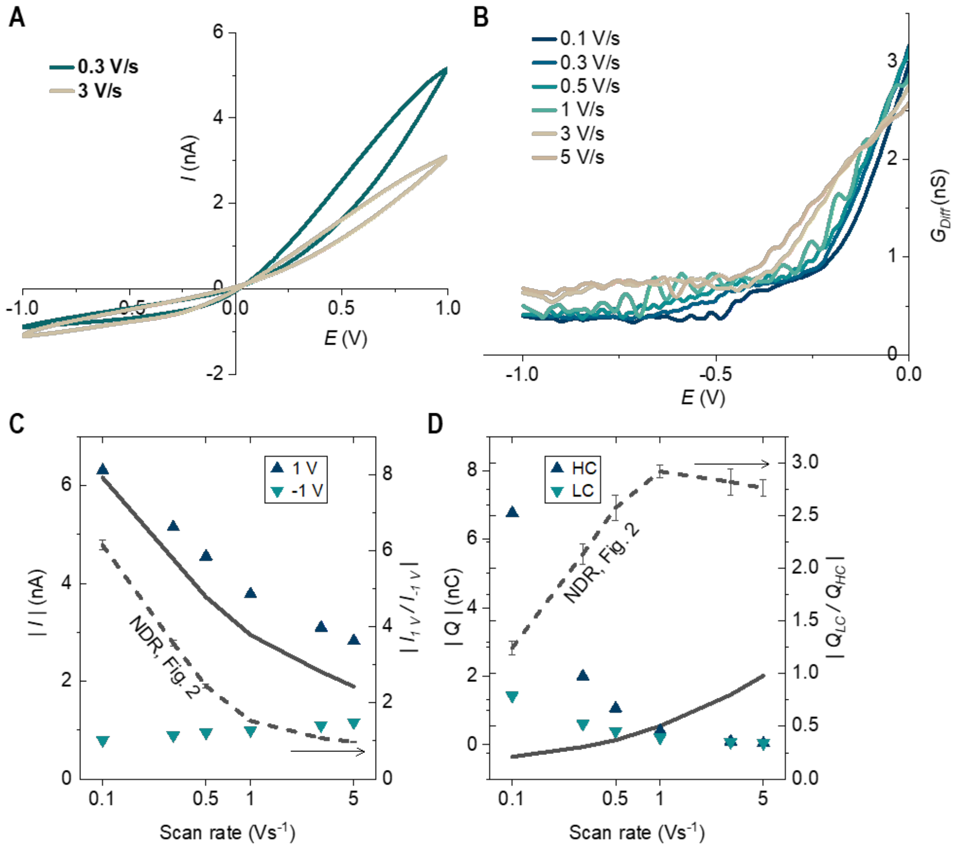

Figure S2. Representative CV, differential conductance, and charge analysis of NPs without NDR. Data from a 200-nm nanopipette in 4 mM KCl. (A) I-V curves with scan rates of 0.3 and 3 V/s. (B) Differential conductance (G_{Diff}) of the backward scans (from positive to negative potentials. (C) ICR and (D) hysteretic charges (<1) over scan rate. Dashed lines are the ratios of ICR and hysteretic charges from the NP with NDR in Figure 2 for comparison, i.e., lower ICR but higher charge ratio (>1).

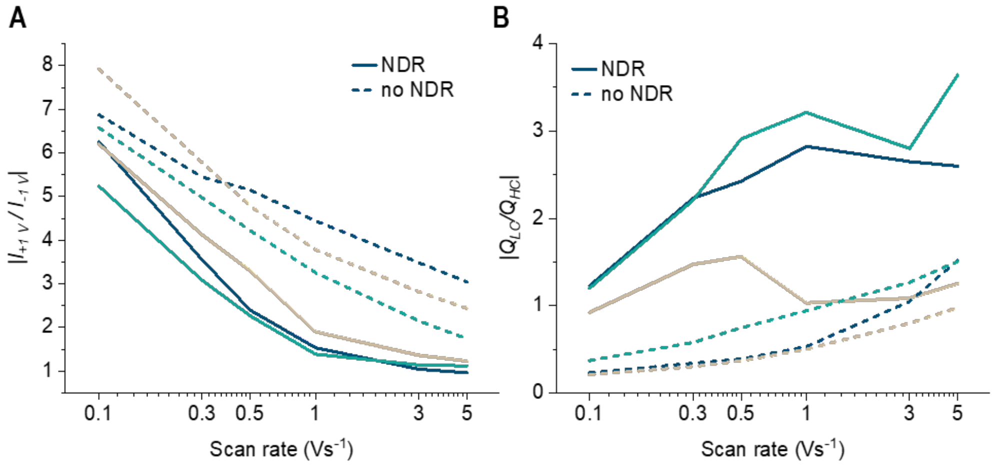

Figure S3. Consistency in the ratios of (A) ICR and (B) hysteretic charges over scan rate from multiple NPs. Solid lines are ICR and charge ratio from three nanopipettes showing NDR, i.e., lower ICR but higher charge ratio, versus dashed lines from three nanopipettes without NDR. The solid indigo lines and dashed beige lines are used in Figures 2 & S2.

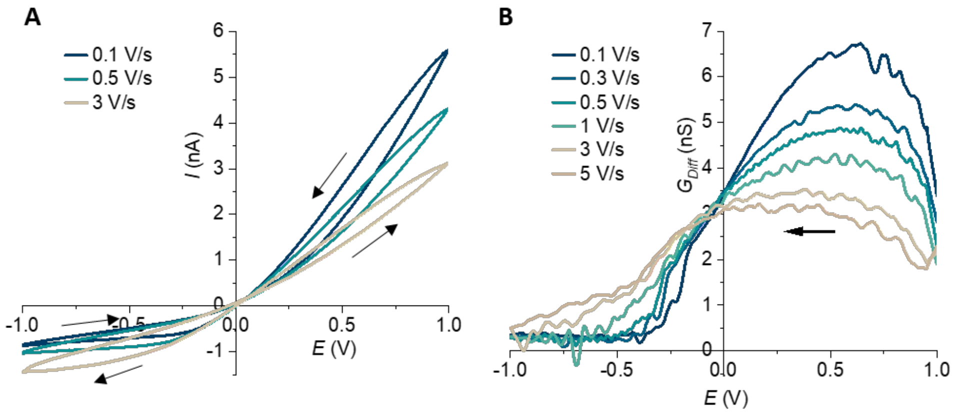

Figure S4. CV data from a 100-nm nanopipette (used in Figures 3A, 3B, 3Ci, and induced abrupt zone) in 4 mM KCl. (A) I-V curves with different scan rates. The potential scan directions are indicated by the arrows. (B) Differential conductance (G_{Diff}) of the backward scans (from positive to negative potentials). No intrinsic NDR was observed without precondition enrichment.

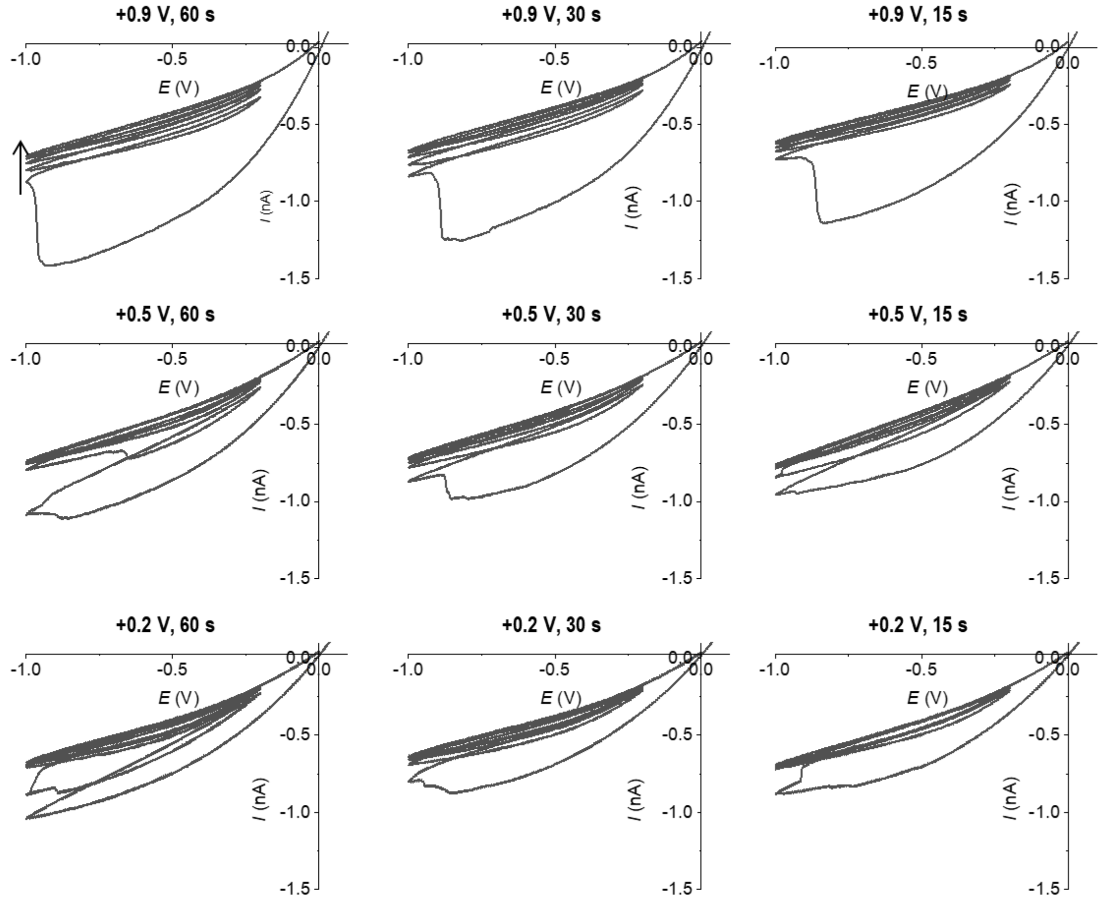

Figure S5. Induced NDR with abrupt transitions. Data from a 100-nm nanopipette (original CV in Fig. S4) in 4 mM KCl solution at 0.1 V/s after the denoted precondition duration and enrichment potential. The sweeping potential was limited with [-0.2 V, -1.0 V] after preconditioning. Arrow indicates the gradual decrease of the +0.9 V, 60 s curve over five scans. Original CV is in Figure S4; Charge and differential conductance analysis are in Figures 3Bii and 3Ci, respectively.

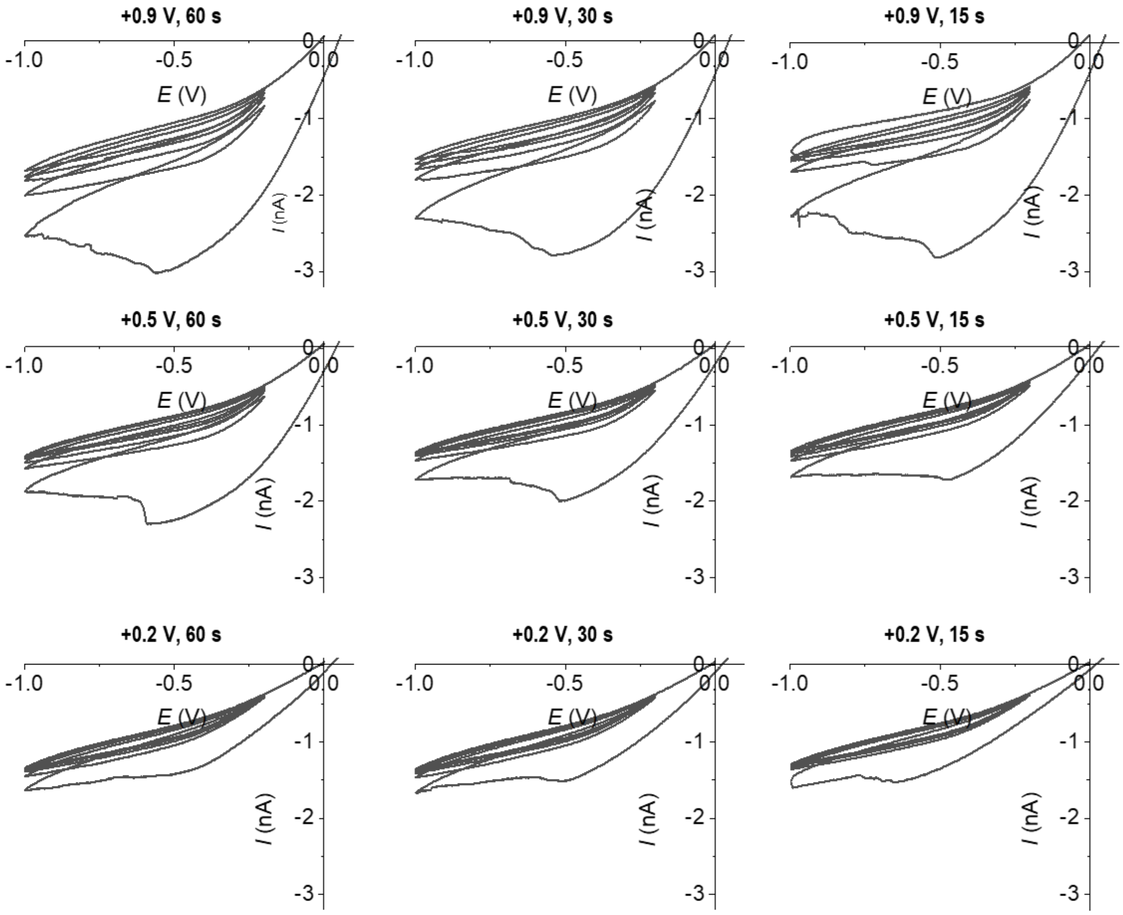

Figure S6. Intrinsic NDR with abrupt transitions. Data from a 50-nm nanopipette in 4 mM KCl solution at 0.1 V/s after the denoted precondition duration and enrichment potential. The sweeping potential was limited with [-0.2 V, -1.0 V] after preconditioning. Original CV and differential conductance analysis are in Figure 2.

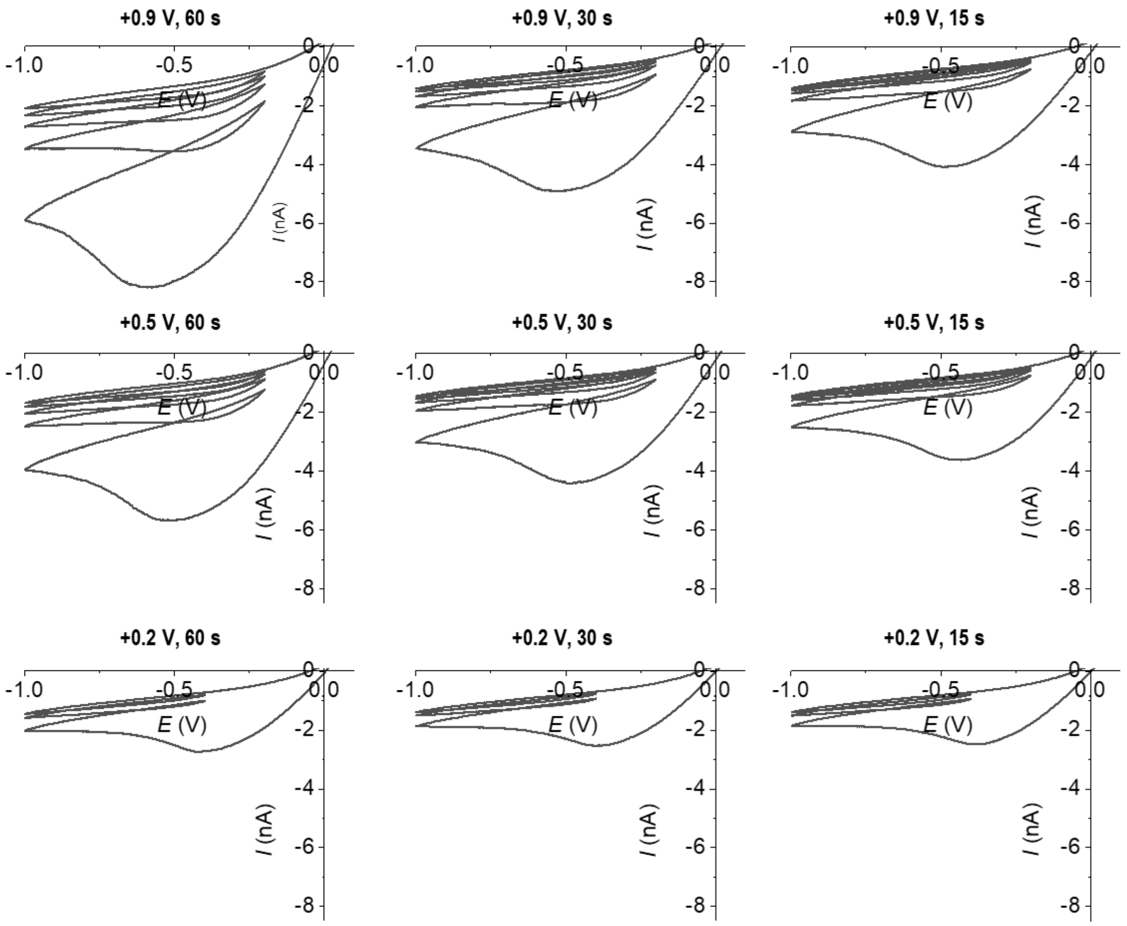

Figure S7. Intrinsic NDR with graduate transitions. Data from a 450-nm nanopipette in 4 mM KCl solution at 0.1 V/s after the denoted precondition duration and enrichment potential. The sweeping potential was limited with [-0.2 V, -1.0 V] after preconditioning. Original CV and differential conductance analysis are in Figure S13.

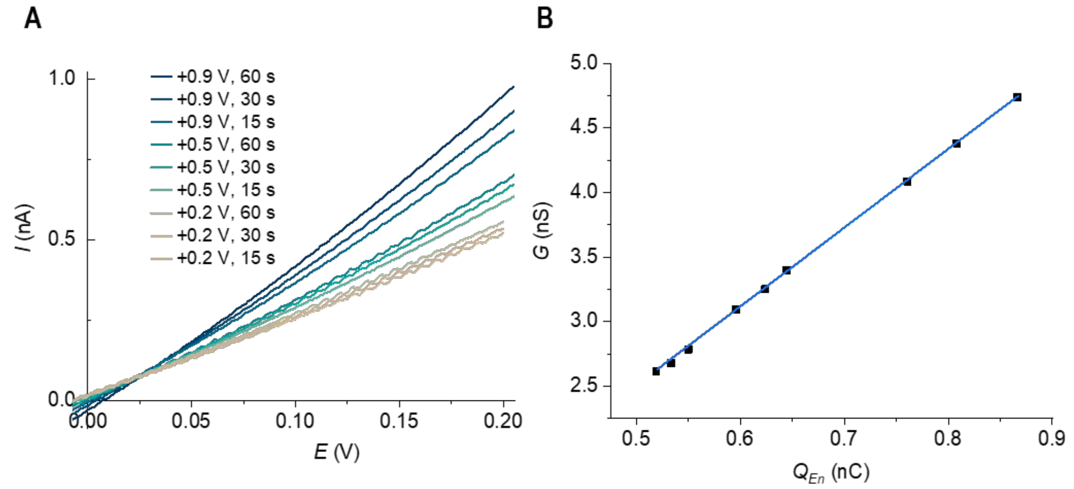

Figure S8. Characterization of the precondition. (A) CV data after the denoted precondition enrichment duration and potential. Data from a 100-nm nanopipette in 4 mM KCl solution at 0.1 V/s (used in Figures 3A, 3B, 3Ci, and induced abrupt zone). (B) Correlation of the conductance at +0.2 V and the enriched hysteretic charges (area under the current curve over scan rate, see Figure 3 for definition). Blue line is the linear regression fitting with R value=0.99. Charge and differential conductance analysis are in Figure 3Bii and 3Ci, respectively.

Note: the integrated charges here also include the through-nanopore flux, thus not the pure hysteretic charges. Since we aim to determine the NDR features within the LC range, there is no complete CV cycles for subtraction as done in Figure 2. The high-quality linear correction with the single point current or conductance, e.g., at +0.2V, validates the Q_{En} as an effective parameter to characterize the extent of the precondition. The integration instead of a single-point readout also reduces the uncertainties from random noise.

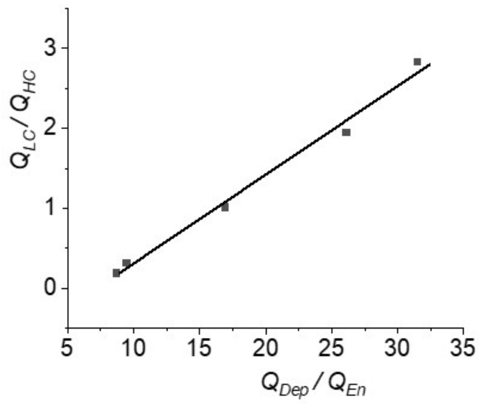

Figure S9. Correlation of the depleted/enriched hysteresis charge Q_{Dep}/Q_{En} *and the LC/HC hysteresis charge loop* Q_{LC}/Q_{HC}. Q_{LC} *and* Q_{HC} *are varied by scan rates in the range of 0.1 to 1 V/s (see Figure 2 for definition);* Q_{Dep} *and* Q_{En} *is varied by precondition enrichment (see Figure 3 for definition).*

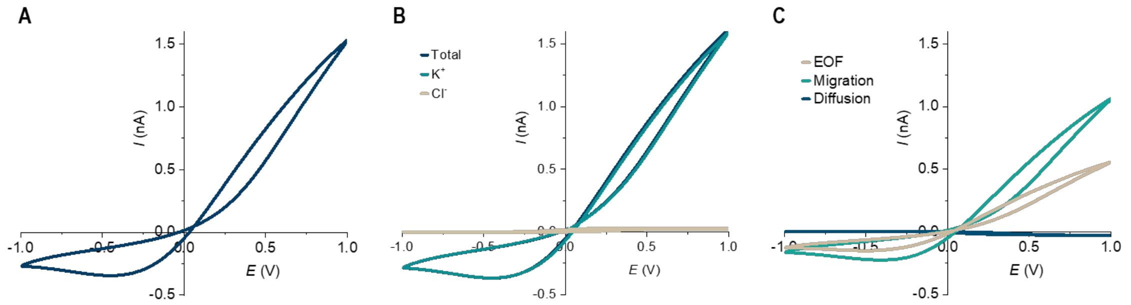

Figure S10. Simulated I-V curves through a 60-nm pore and a half cone angle of 5° in 1 mM KCl solution. (A) I-V *curve. (B) The contribution of cation and anion to the total flux. (C) The contribution of EOF, migration and diffusion to the total cation flux.*

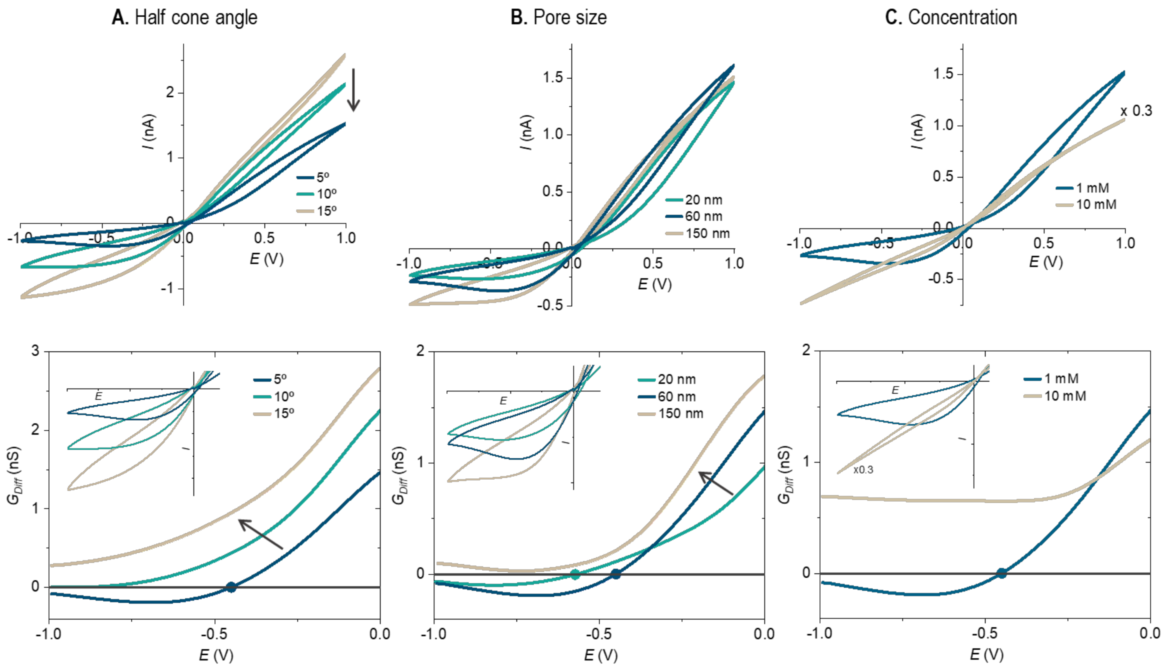

Figure S11. Simulated I-V curves with nanogeometry and measurement parameter variations. The top panels are the simulated I-V curves and the corresponding bottom ones are differential conductance (GDiff) of the backward scans after enrichment. (A) A 60-nm nanopore with different half cone angles in 1 mM KCl; (B) different tip radius with a half cone angle of 5° in 1 mM KCl; (C) a 60-nm-radius nanopore with a half cone angle of 5° in different KCl concentrations.

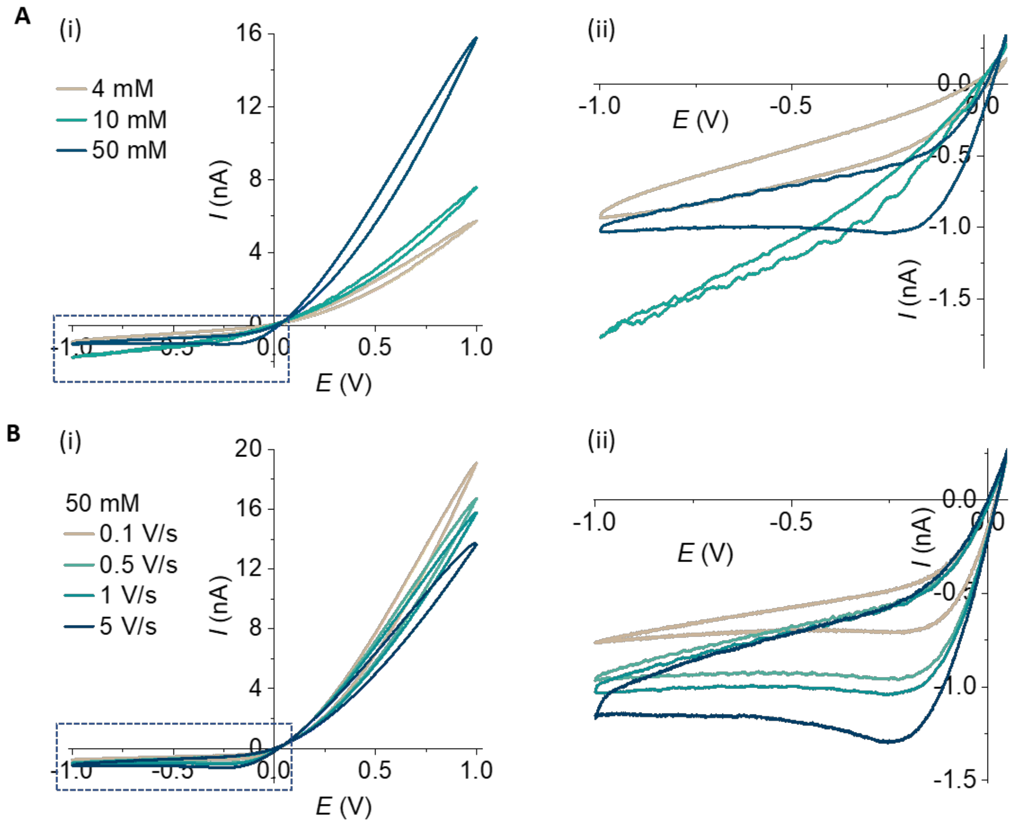

Figure S12. CV data from a 100-nm nanopipette showing NDR in 50 mM KCl. (A) I-V curves with different KCl concentrations at 1 V/s. (B) I-V curves with different scan rates in 50 mM KCl. Panels (ii) present a magnified view of the box area highlighted in panels (i).

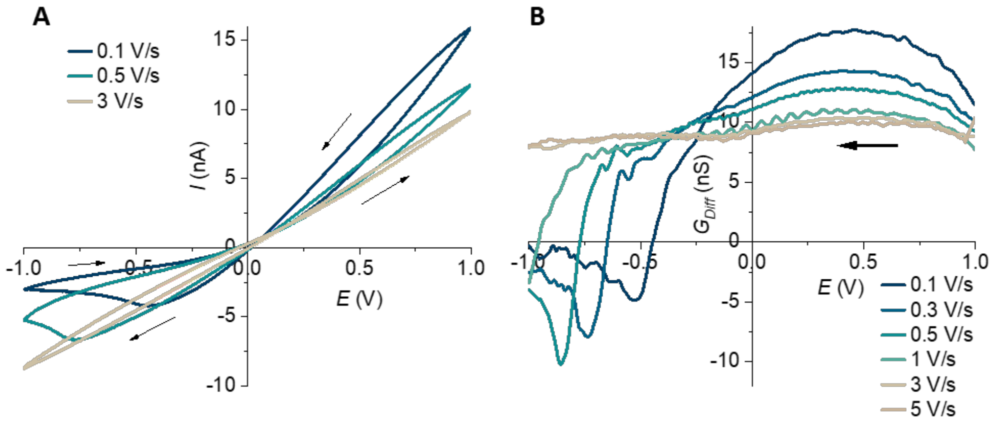

Figure S13. CV data from a 450-nm nanopipette (used in Figure 3Cii and intrinsic gradient zone) in 4 mM KCl. (A) I-V curves with different scan rates. The potential scan directions are indicated by the arrows. (B) Differential conductance (G_{Diff}) of the backward scans (from positive to negative potentials). The potential at G_{Diff} =0 corresponds to NDR peak potential (V_{NDR}).

References:

1. Yu, R.-J.; Ying, Y.-L.; Gao, R.; Long, Y.-T., Confined Nanopipette Sensing: From Single Molecules, Single Nanoparticles, to Single Cells. *Angew. Chem. Int. Ed. Engl.* **2019,** *58* (12), 3706-3714.
2. Brown, W.; Kvetny, M.; Yang, R.; Wang, G., Selective Ion Enrichment and Charge Storage through Transport Hysteresis in Conical Nanopipettes. *J. Phys. Chem. C* **2022,** *126* (26), 10872-10879.
3. Brown, W.; Kvetny, M.; Yang, R.; Wang, G., Higher Ion Selectivity with Lower Energy Usage Promoted by Electro-osmotic Flow in the Transport through Conical Nanopores. *J. Phys. Chem. C* **2021,** *125* (6), 3269-3276.
4. Wang, D.; Liu, J.; Kvetny, M.; Li, Y.; Brown, W.; Wang, G., Physical origin of dynamic ion transport features through single conical nanopores at different bias frequencies. *Chem. Sci.* **2014,** *5* (5), 1827-1832.
5. Liu, J.; Kvetny, M.; Feng, J.; Wang, D.; Wu, B.; Brown, W.; Wang, G., Surface Charge Density Determination of Single Conical Nanopores Based on Normalized Ion Current Rectification. *Langmuir* **2012,** *28* (2), 1588-1595.
6. Liu, J.; Wang, D.; Kvetny, M.; Brown, W.; Li, Y.; Wang, G., Quantification of Steady-State Ion Transport through Single Conical Nanopores and a Nonuniform Distribution of Surface Charges. *Langmuir* **2013,** *29* (27), 8743-8752.
